# Group B *Streptococcus* colonization at delivery is associated with maternal peripartum infection

**DOI:** 10.1371/journal.pone.0264309

**Published:** 2022-04-01

**Authors:** Anne Karin Brigtsen, Anne Flem Jacobsen, Lumnije Dedi, Kjetil Klaveness Melby, Cathrine Nygaard Espeland, Drude Fugelseth, Andrew Whitelaw

**Affiliations:** 1 Institute of Clinical Medicine, University of Oslo, Oslo, Norway; 2 Department of Neonatal Intensive Care, Oslo University Hospital Ullevål, Oslo, Norway; 3 Department of Obstetrics and Gynecology, Oslo University Hospital Ullevål, Oslo, Norway; 4 Department of Microbiology, Oslo University Hospital Ullevål, Oslo, Norway; Universidade de Lisboa Faculdade de Medicina, PORTUGAL

## Abstract

**Background:**

Group B *Streptococcus* (GBS) is a major cause of serious neonatal infection but its role in maternal morbidity has received little investigation. The aim of this study was to determine whether GBS colonization at delivery is associated with increased risk of maternal peripartum infection.

**Methods:**

In this prospective cohort study, 1746 unselected women had a vaginal-rectal culture taken at the onset of labor. Diagnosis of maternal peripartum infection was based on a combination of two or more signs or symptoms including fever, breast pain, severe wound or pelvic pain, purulent discharge and abnormal laboratory tests including C-reactive protein and white blood cell count occurring from labor until 2 weeks postpartum. The main outcome measure was the proportion of women with maternal peripartum infection according to GBS colonization status.

**Results:**

A total of 25.9% (452/1746) women were colonized with GBS. The rate of peripartum infection was almost twice as high in colonized women (49/452 [10.8%]) vs. non-colonized women (81/1294 [6.3%]); OR 1.82 [1.26–2.64], p = 0.002). This association was confirmed in a multivariable model (OR 1.99 [1.35–2.95], p = 0.001). Women diagnosed with peripartum infection had a significantly longer hospital stay compared to women without peripartum infection (4 days (median) vs. 3 days, p < 0.001). Length of hospital stay did not differ between colonized and non-colonized women. Serotype IV GBS was more frequent in colonized women with peripartum infection than in women without peripartum infection (29.3% vs. 12.5%, p = 0.003).

**Conclusions:**

GBS colonization at delivery is associated with increased risk of peripartum infection. Whether this increase is due directly to invasion by GBS or whether GBS colonization is associated with a more general vulnerability to infection remains to be determined.

## Introduction

Group B *Streptococcus* (GBS, *Streptococcus agalactiae*) colonizes the urogenital or gastrointestinal tract in 10–30% of women giving birth [1]. GBS rarely leads to invasive maternal disease, but it is a dominant cause of morbidity and mortality in infants, leading to early- and late-onset GBS sepsis, meningitis and pneumonia [2]. Maternal colonization at delivery is considered the main risk factor for early-onset neonatal GBS disease [2]. Many high-income countries, such as the US, Spain and Germany, use vaginal-rectal culture screening at 35–38 weeks of gestation to identify GBS colonization. A positive culture warrants the use of intrapartum antibiotic prophylaxis to prevent early-onset neonatal GBS disease [1,3–7]. Other high-income countries, like Norway, UK and the Netherlands, have a risk-based strategy, giving intrapartum antibiotic prophylaxis to women in labor with antenatal GBS bacteriuria, preterm delivery, intrapartum fever, prelabor rupture of membranes (PROM), and in those with a previous GBS infected infant [8,9]. Additional risk factors for maternal GBS colonization have been reported, such as black race, low parity, high age and high body mass index (BMI), but whether these risk factors are universal or restricted to certain geographic areas remain unclear [10,11].

While the emphasis of prior research has been on the serious consequences of neonatal infection, there is less information on maternal complications associated with GBS colonization [12,13]. A reduced occurrence of chorioamnionitis has been reported from two studies in U.S. populations after the introduction of a screening-based neonatal GBS disease prevention strategy [14,15]. However, in populations with a risk-based strategy, where the use of intrapartum antibiotics is likely lower than in populations using a screening-based strategy, there is sparse information about peripartum infection. Accordingly, the primary objective of this study was to determine whether GBS colonization at delivery was associated with increased risk of peripartum infection. The secondary objective was to identify risk factors for GBS colonization at delivery in a large, contemporary, unselected population.

## Material and methods

The Oslo GBS Study is a prospective cohort study of pregnant women admitted to the Delivery department at Oslo University Hospital Ullevål, Oslo, Norway. This is the second largest delivery department in Scandinavia with more than 7000 deliveries per year, serving a population of approximately 600 000 in the metropolitan Oslo area. Between June 2009 and September 2011, 16 000 pregnant women were consecutively invited to participate at the time of ultrasound screening in the second trimester. We excluded women who were likely to terminate the pregnancy due to lethal fetal anomaly. This was expected to account for <0.5% of the pregnancies [16]. The consent form was in Norwegian, limiting the population to women with adequate knowledge of the Norwegian language. A total of 4450 women consented, out of whom 1746 had one vaginal-rectal sample obtained at delivery. Sample size calculation was based on the following assumptions: an alpha (risk of type I error) of 5%, a power (1-beta (risk of type II error)) of 90% and a maternal GBS colonization rate of 25%. With these assumptions, detecting a difference of 5% compared with 10% in the rate of the primary outcome would require a total sample of 1596.

Midwives collected combined vaginal-rectal swabs during the vaginal examination at the admission to labor ward and before administration of antibiotics. Hospital staff taking care of the women during and after delivery were blinded to the results of the GBS cultures. Collection and processing of the swabs were performed according to the CDC recommendations, as previously described [17,18]. In brief, the swabs were placed in transport tubes containing Amies medium (Copan, Brescia, Italy) and transported to the laboratory within 24 hours. Each strain was cultured to both Columbia agar (Becton, Dickinson and Company, Franklin Lakes, New Jersey, US) and inoculated into a selective enrichment broth (Lim broth, Becton, Dickinson and Company, Franklin Lakes, New Jersey, US). After incubation at 35°C in ambient air for 24 hours, turbid broths were subcultured onto blood agar plates and incubated at 35°C in 5% CO_2_ for 24 hours. Columbia agar plates were reincubated at 35°C for 18–24 hours if GBS was not identified after incubation. Isolates were identified based on colony morphology, ß-hemolysis, catalase test, and latex agglutination. The Lancefield group B antigen was determined using a latex agglutination grouping kit (Prolex, Prolab Diagnostics, Richmond Hill, Ontario, Canada), according to the manufacturer’s recommendations.

Clinical, demographic and epidemiological information were obtained from medical records, the Medical Birth Registry of Norway and the Norwegian Surveillance System of Communicable Diseases. The Medical Birth Registry of Norway prospectively records data on all births in Norway based on compulsory notification.

The patient records of all participating women were systematically examined, and a diagnosis of maternal peripartum infection was made on the basis of a combination of two or more of the following signs and symptoms including rectal temperature ≥38.0°C, breast pain, severe wound or pelvic pain, purulent discharge, and abnormal laboratory tests including increased C-reactive protein and/or white blood cell count according to pregnancy references [19]. The peripartum period was defined as during labor and/or within 2 weeks after delivery. This is within the span the mothers have to contact the hospital if they experience signs or symptoms of infection or other conditions related to delivery. Obstetrical interventions encompassed any one of the following: amniotomy, the use of forceps, vacuum extraction, caesarean delivery, manual removal of the placenta or retained placental tissue, uterine curettage, and episiotomy. PROM was defined as rupture of membranes ≥18 hours before delivery. Antibiotic exposure included complete (≥ 4 hours before delivery) or incomplete (< 4 hours before delivery) intrapartum antibiotic prophylaxis, prophylaxis due to emergency cesarean delivery or treatment of maternal infection at any time during the peripartum period as defined above.

Baseline characteristics are reported as counts and percentages for categorical variables. For normally distributed continuous variables, we report means and standard deviations. For variables with skewed distributions, we report medians and interquartile ranges. Chi-square or Fisher’s exact tests, as appropriate, were used to compare categorical variables between GBS colonized and non-colonized women. Concerning continuous variables, Student’s *t*-test or the Mann-Whitney *U*-test were used to compare between-group differences. We used logistic regression analysis to assess the association between potential risk factors and maternal GBS colonization as well as the association between the primary predictive variable GBS colonization and the primary outcome variable, maternal peripartum infection. Potential confounders of this association included gestational age, PROM, obstetrical interventions, cesarean delivery, maternal age, BMI, maternal illness before pregnancy, diabetes mellitus, asthma, maternal bleeding in pregnancy, recurring urinary tract infection, discolored amniotic fluid, giving birth to twins, and infant weight. We present risk estimates as odds ratios (OR) with 95% confidence interval (CI). A two-sided *p*-value < 0.05 was considered to indicate statistical significance. Rates of missing data were generally low, and actual numbers are provided in Table 1. The IBM SPSS software, version 22, was used for all analyses.

**Table 1:** Characteristics of GBS colonized and non-colonized women.

Characteristic	Colonized women (*n* = 452)	Non-colonized women (*n* = 1294)	*p*-value
Maternal age (years)	32.0 ± 3.9	32.3 ± 4.1	0.25
Maternal age > 40 years	8 (1.8)	45 (3.5)	0.07
Married or cohabitant			
No. of participants/total no. (%)	440/450 (97.8)	1247/1289 (96.7)	0.27
Parity			
Nulliparous			
No. of participants/total no. (%)	283/450 (62.9)	816/1290 (63.3)	0.89
Primiparous			
No. of participants/total no. (%)	128/450 (28.4)	373/1290 (28.9)	0.85
Multiparous			
No. of participants/total no. (%)	39/450 (8.7)	101/1290 (7.8)	0.57
Ethnicity			
Norwegian	387 (86.2)	1090 (84.4)	0.37
Nordic	403 (89.2)	1132 (87.5)	0.35
Weight (kg)	65.8 ± 10.8	65.8 ± 10.9	0.98
Weight gain (kg)	14.1 ± 4.6	14.3 ± 4.9	0.47
Height (cm)	168.4 ± 6.0	168.2 ± 6.0	0.60
BMI (kg/m^2^)			
<18.5			
No. of participants/total no. (%)	9/404 (2.2)	31/1161 (2.7)	0.63
18.5–24.9			
No. of participants/total no. (%)	292/404 (72.3)	839/1161 (72.3)	>0.99
25–29.9			
No. of participants/total no. (%)	84/404 (20.8)	225/1161 (19.4)	0.54
30–39.9			
No. of participants/total no. (%)	17/404 (4.2)	62/1161 (5.3)	0.37
≥40			
No. of participants/total no. (%)	2/404 (0.5)	4/1161 (0.3)	0.65
BMI change (kg/m^2^)	5.0 ± 1.6	5.1 ± 1.7	0.39
Diabetes mellitus^a^	4 (0.9)	15 (1.2)	0.63
Asthma	16 (3.7)	28 (2.2)	0.10
Disease^b^	44 (9.9)	106 (8.5)	0.36
Pre-eclampsia/eclampsia	4 (0.9)	13 (1.0)	>0.99
PROM^c^	65 (14.4)	189 (14.6)	0.73
Meconium-stained amniotic fluid	48 (11.0)	154 (12.3)	0.47
Amniotomy			
No. of participants/total no. (%)	34/434 (7.8)	93/1254 (7.4)	0.78
Induction using Foley bulb			
No. of participants/total no. (%)	2/434 (0.5)	13/1254 (1.0)	0.38
Intervention^d^	195 (44.2)	551 (43.6)	0.83
Emergency cesarean delivery	50 (11.4)	127 (10.0)	0.40
Vacuum extraction			
No. of participants/total no. (%)	55/434 (12.7)	184/1257 (14.6)	0.31
Forceps	4 (0.9)	12 (0.9)	0.94
Epidural anesthesia			
No. of participants/total no. (%) Incomplete IAP^e^ No. of participants/total no. (%) Complete IAP^f^ No of participants/total no. (%) Antibiotics during delivery^g^ No of participants/total no. (%)	168/438 (38.4) 21 (4.6) 29/452 (6.4) 85/452 (18.8%)	567/1265 (44.8) 50/1290 (3.9) 53/1290 (4.1) 190/1293 (14.7)	0.02 0.48 0.046 0.04
Length of hospitalization—days	3.3 ± 1.4	3.3 ± 1.3	0.88

Values are mean ± standard deviation or n (%).

^a^Diabetes mellitus, includes type I, type II, gestational, unspecified diabetes mellitus and the use of antidiabetic medication.

^b^Disease includes asthma, urinary tract infection, renal disease, hypertension, rheumatoid arthritis, heart disease, epilepsy, thyroid disease and diabetes mellitus prior to pregnancy.

^c^PROM, prelabor rupture of membranes (≥18 hours).

^d^Intervention, amniotomy, the use of forceps, vacuum extraction, cesarean delivery, manual removal of the placenta or retained placental tissue, uterine curettage or episiotomy.

^e^Incomplete IAP, intrapartum antibiotic prophylaxis < 4 hours before delivery.

^f^Complete IAP, intrapartum antibiotic prophylaxis ≥ 4 hours before delivery.

^g^Antibiotics during delivery including complete or incomplete intrapartum antibiotic prophylaxis, prophylaxis due to emergency cesarean delivery, PROM or treatment of maternal infection.

The study was approved by the Regional Committee for Medical and Health Research Ethics South East (reference number 6.2008.1615), and the hospital’s Data Protection Officer prior to commencement and was registered in the Norwegian Biobank Registry. Written informed consent was obtained from the participants.

## Results

A total of 1746 consenting women in whom a vaginal-rectal sample was obtained, were included in the present analysis. Vaginal-rectal colonization with GBS was present in 452 (25.9%) of the 1746 sampled women. Baseline characteristics of the mothers stratified according to colonization status are summarized in Table 1. The age ranged from 19.7 to 45.7 years for colonized and from 19.8 to 46.2 years for non-colonized women. The mean age did not differ between colonized and non-colonized women (32.0 vs. 32.3 years, p = 0.25). The colonization rate tended, however, to be lower among women > 40 years than in women ≤ 40 years (8 of 53 (15.1%) vs. 444 of 1693 (26.2%), p = 0.07). The rate of colonized versus non-colonized did not differ between mothers born in compared with outside Norway, nulliparous compared to multiparous, or married or cohabitant compared to single mothers. Regarding maternal comorbidities, there were no significant differences concerning the prevalence of diabetes mellitus (i.e., type I, type II, gestational or unspecified diabetes) or asthma. Likewise, there were no significant differences with regard to the incidence of pre-eclampsia/eclampsia, or PROM. GBS colonized women were not prone to more obstetrical interventions, which included the use of forceps, vacuum extraction, cesarean delivery, manual removal of placental tissue, uterine curettage or episiotomy, than non-colonized women. A total of 739 (42.3%) women provided information concerning smoking habits. The frequency of current smoking was similar in colonized and non-colonized women (12/197 [6.1%] vs. 41/542 [7.6%], p *=* 0.49).

In total, 130 women were diagnosed with peripartum infection. Mastitis (n = 43) and chorioamnionitis (n = 34) were the two most common types of infection (S1 Table). The odds ratios for infection based on pregnancy characteristics are shown in Table 2. A total of 49 (10.8%) out of 452 women colonized with GBS compared with 81 (6.3%) of 1294 non-colonized women were diagnosed with peripartum infection. Thus, the odds of peripartum infection were almost twice as high for colonized women compared with non-colonized women (OR 1.82, 95% CI: 1.26–2.64, p = 0.002).

**Table 2:** Unadjusted and adjusted odds ratios for maternal peripartum infection.

Variable	Unadjusted OR (95% CI)^a^	*p*-value	Adjusted OR (95% CI)^b^	*p*-value
Colonization	1.82 (1.26–2.64)	0.002	1.99 (1.35–2.95)	0.001
Gestational age (per week)	0.89 (0.81–0.96)	0.004	0.91 (0.83–1.00)	0.049
PROM^c^	2.45 (1.70–3.53)	<0.001	2.31 (1.55–3.45)	<0.001
Intervention	3.16 (2.13–4.68)	<0.001	2.89 (1.89–4.42)	<0.001
Antibiotics due to emergency cesarean delivery Cesarean delivery Vacuum extraction Forceps Amniotomy	1.94 (1.11–3.39) 3.36 (1.66–6.83) 2.02 (1.30–3.14) 0.83 (0.11–6.31) 0.98 (0.48–1.98)	0.02 0.001 0.002 0.89 0.95	1.37 (0.75–2.51)	0.31
Maternal age (per year)	0.99 (0.96–1.03)	0.64		
BMI (per kg/m^2^)	1.01 (0.97–1.07)	0.45		
Maternal illness before pregnancy	1.38 (0.77–2.47)	0.29		
Diabetes mellitus	0.69 (0.04–5.20)	0.72		
Asthma	1.31 (0.46–3.71)	0.62		
Maternal bleeding in pregnancy	4.36 (0.45–42.22)	0.20		
Recurring urinary tract infection	0.55 (0.07–4.14)	0.56		
Discolored amniotic fluid	1.42 (0.85–2.38)	0.18		
Giving birth to twins	1.40 (0.46–6.10)	0.65		
Infant weight (kg)	0.79 (0.59–1.12)	0.18		

^a^OR, odds ratios; CI, confidence interval.

^b^Adjusted for gestational age, PROM, interventions, and antibiotics due to emergency cesarean delivery.

^c^PROM, prelabor rupture of membranes (≥18 hours).

Obstetrical interventions were common and occurred in 746 (43.8%) of the 1704 deliveries from which data were available. Interventions such as amniotomy, the use of forceps, vacuum extraction, cesarean delivery, manual removal of the placenta or retained placental tissue, uterine curettage and episiotomy were associated with more than three times increased risk of peripartum infection (OR 3.16, 95% CI: 2.13–4.68, p < 0.001). Considering cesarean delivery in isolation, the OR point estimate was even higher, but the confidence interval wider (OR 3.36, 95% CI: 1.66–6.83). Considering vacuum extraction, the point estimate was considerably lower than for the variable obstetrical intervention. Accordingly, because of the overlap between these three variables, in multivariable analyses the variable obstetrical intervention rather than cesarean delivery or vacuum extraction was used. Gestational age, PROM, and antibiotics due cesarean delivery were also significantly associated with the risk of peripartum infection in unadjusted analyses.

After adjusting for gestational age, PROM, obstetrical interventions, and antibiotics due to emergency cesarean delivery in a multivariable model, the risk of peripartum infection in colonized women was basically unchanged (OR 1.99, 95% CI: 1.35–2.95, p = 0.001). In the multivariable model, the risk of peripartum infection was still significantly increased for women diagnosed with PROM, in those having obstetrical interventions, and those giving birth to infants with lower gestational age (Table 2). In contrast, the association between antibiotics due to emergency cesarean delivery and peripartum infection in colonized women was attenuated and no longer significant.

Women diagnosed with peripartum infection (n = 130) had a longer hospital stay than women without peripartum infection (n = 1613) (4 days (median) vs. 3 days, p < 0.001).

Despite an increased risk of peripartum infection, colonized women did not have a longer hospital stay than non-colonized women (Table 1).

Out of the 452 colonized women, serotype data were available for 426 (94.2%), and for colonized women with peripartum infection, serotype data was available for 41 of 49 (83.7%). For serotypes Ia, Ib, II and III there were no significant difference in the serotype distribution between women with and without peripartum infection (Table 3). In contrast, colonization with serotype IV was over-represented among women with peripartum infection compared to women without peripartum infection (12/41 [29.3%] compared with 48/385 [12.5%], p = 0.003), while serotype V was less frequent in women diagnosed with peripartum infection (2/41 [4.9%] vs. 70/385 [18.2%], p = 0.03).

**Table 3:** Serotype distribution in women with and without peripartum infection.

Serotype	Women without peripartum infection (*n* = 385)	Women with peripartum infection (*n* = 41)	*p*-value
**Ia**	57 (14.8)	10 (24.4)	0.11
**Ib**	39 (10.1)	3 (7.3)	0.78
**II**	57 (14.8)	2 (4.9)	0.08
**III**	94 (24.9)	12 (29.3)	0.49
**IV**	48 (12.5)	12 (29.3)	0.003
**V**	70 (18.2)	2 (4.9)	0.03
**VI**	2 (0.5)		
**VII**	0 (0.0)		
**VIII**	3 (0.8)		
**IX**	12 (3.1)		
**NT**	3 (0.8)		

Values are n (%).

## Discussion

This large-scale, prospective cohort study demonstrates that women colonized with GBS at the time of delivery were almost twice as likely to acquire a peripartum infection compared to non-colonized women. The increased risk was independent of gestational age and interventions, such as cesarean delivery. Although peripartum infection lengthened hospital stay, overall, colonized women did not have an extended length of stay compared to non-colonized women.

There are at least two possible explanations for the association between maternal GBS colonization and peripartum infection. Maternal colonization with GBS has the potential to evolve into both local and systemic infection [20]. Although the exact pathophysiological mechanisms responsible for maternal infections with GBS remain uncertain, data suggest that specific GBS virulence factors may play a role. Thus, the hypervirulent GBS adhesin (HvgA) expressed by the GBS ST17 clone is associated with late-onset sepsis in the newborn and is likely to play a role in the crossing of the mucosal barrier also in the mother [21]. The HvgA is critical for gut colonization and bacterial crossing of the intestinal and blood-brain barriers in infants [22]. Other GBS virulence factors such as pili and toxins may also contribute to the pathogenesis [23,24]. In addition, GBS colonization may be associated with a general impairment of defense against microbes. GBS is a commensal bacterium, and in pregnant women colonized with GBS, it has been shown that vaginal epithelial cells change properties leading to alterations in vaginal immunity [25]. This may increase the susceptibility to colonization and infections with other pathogens. In a study by Andrews et al., GBS-positive women in the third trimester were more frequently colonized with *Staphylococcus aureus* than GBS-negative women [26]. Furthermore, as there are variable patterns in maternal antibodies to GBS, women with peripartum infection may have less type-specific antibodies directed towards the colonizing GBS serotype, making them more prone to peripartum infection.

Despite the awareness of maternal GBS colonization during delivery, few studies have examined the consequence this may have on peripartum infection. In a study by Krohn et al., GBS colonization was associated with an increased risk of postpartum endometritis [13]. Heavy GBS colonization was associated with intra-amniotic infection, whereas light colonization was not. Yancey et al. noted an increased risk of chorioamnionitis, but not endometritis, with increasing vaginal GBS bacterial load [27]. Both studies were performed prior to the recommendations of intrapartum antibiotic prophylaxis to prevent perinatal GBS disease [28]. Two more recent studies have looked at maternal peripartum infection in populations with a screening-based strategy to prevent neonatal GBS infection [14,15]. In a single center historical cohort study including 60 029 pregnant women from 2003 to 2015, GBS colonization at any time point during the pregnancy was associated with decreased incidence of chorioamnionitis, and wound infection [14]. In a retrospective study, including 170 804 term deliveries of women enrolled between 2002 and 2008, GBS colonization was associated with slightly lower odds of chorioamnionitis [15]. In contrast to our study that was performed in a country with a risk-based prevention strategy, they did not find an association with postpartum infection. Taken together, these findings suggest that routine administration of intrapartum antibiotics to all GBS colonized women may be associated with reduced risk of maternal peripartum infection. However, as we used a risk-based strategy for intrapartum neonatal prophylaxis, this could not be assessed in our cohort.

The association between obstetric interventions and maternal peripartum infections are well established [29,30]. In the study by Minkoff et al. in 1980, GBS vaginally colonized patients who underwent cesarean delivery had a higher incidence of fever, endometritis, use of antibiotics, and prelabor rupture of membranes [12]. In contrast, we found the risk of peripartum infection in GBS colonized women to be independent of interventions such as cesarean delivery. Improvement in surgical techniques and anesthesia, shorter duration of surgery, improved hygiene, faster mobilization, shorter hospital stay and prophylactic antibiotic to patients with acute cesarean delivery may explain some of this difference.

In our study, the GBS colonized and non-colonized women were quite similar with respect to epidemiological and clinical characteristics. Our data showed a nonsignificant trend towards decreased colonization rates in older mothers. Moreover, we did not find differences in GBS colonization rates, neither across BMI strata nor according to maternal ethnicity. These observations are in contrast to findings in some previous studies, in which older mothers, high BMI, black race and low parity have been associated with GBS colonization [10,11,31]. Reasons for these apparent discrepancies may include geographical disparities in risk factor profiles and demographics as well as the different range in socioeconomic status between countries. For instance, teen pregnancies are more common, and the BMI of premenopausal women is markedly higher in the US than in the Scandinavian countries [32–35]. In addition, prevention strategies for maternal GBS colonization may differ between countries. Accordingly, our findings underscore the point that findings from a specific geographical region may not be extrapolated to other countries or continents.

Serotype IV was associated with increased risk of maternal peripartum infection. This result is in accordance with previous studies reporting serotype IV as an emerging serotype both in early-onset GBS disease, adult invasive disease and in GBS colonized women [36–39].

Our study has several strengths, including the prospective design and the inclusion of women from a predominantly unselected population. The blinded design represents an additional quality in that the maternal GBS colonization status was not available to the medical personnel, and consequently did not influence their clinical decisions. Conversely, the study also has notable limitations. First, the consent form was in Norwegian, which likely resulted in a selection bias in the study population. Approximately 30% of women giving birth at Oslo University Hospital are of Asian, African, Central or South-American origin, whereas they accounted for 6.3% of the study population. In general, they are less familiar with the Norwegian language and fewer have education after primary school than Norwegian women [40]. Thus, this may have skewed the inclusion towards well-educated women familiar with the Norwegian language and contributed to a lower overall inclusion rate. Second, the selection of consenting participants may not completely represent the whole population of women at delivery. Third, in a considerable proportion of women who had provided informed consent to participate in the study the midwives were unable to collect vaginal-rectal specimens, the predominant reason being the high workload in the delivery ward. We believe the likelihood that this has introduced a systematic bias is minor. Finally, this study did not systematically assess other pathogens responsible for peripartum maternal infection. Group A *Streptococcus*, *Escherichia coli* and *Staphylococcus aureus* are common pathogens in the peripartum period which, in addition to GBS, are known to give rise to sepsis, urinary tract infections, wound and breast infections [41–43]. Accordingly, we cannot rule out the possibility that other pathogens may have contributed to the incidence of peripartum maternal infections in our study and that GBS colonization is associated with a more general vulnerability to infection.

Our findings may be of importance for clinicians by rising the awareness of the increased risk of peripartum infection in GBS colonized women compared to non-colonized women. In addition, our findings demonstrate that the burden of GBS disease is higher than previously thought and provides further support for developing a vaccine to target this pathogen.

## Supporting information

S1 Table Types of infection. (DOCX)

S1 Data (XLSX)

## Abbreviations

BMI: body mass index
CDC: Centers for Disease Control and Prevention
CI: confidence interval
GBS: Group B *Streptococcus*
HvgA: hypervirulent GBS adhesin
OR: odds ratios
PROM: prelabor rupture of membranes
