# Supplementary material for: Group B Streptococcus colonization at delivery is associated with maternal peripartum infection
Source: PLoS One. 2022 Apr 1;17(4):e0264309. doi: 10.1371/journal.pone.0264309 (PMC8975154; doi:10.1371/journal.pone.0264309)
Supplement: S1 Table — (DOCX) [file pone.0264309.s001.docx]

**S1 Table. Types of infection**

| **Type of infection** | **Colonized women**  **(n=49)** | **Non-colonized women**  **(n=81)** |
| --- | --- | --- |
|  |  |  |
| Sepsis, GBS | 1 | 0 |
| Sepsis, other pathogens | 1 | 1 |
| Urinary tract infection | 3 | 11 |
| Mastitis | 16 | 27 |
| Endometritis | 0 | 9 |
| Chorioamnionitis | 15 | 19 |
| Wound infection | 3 | 6 |
| Pneumonia | 0 | 1 |
| Genital herpes | 1 | 0 |
| Clinical infection, not specified | 9 | 7 |
